# Risk factors and prevalence of work-related injuries and accidents among veterinarians in India

**DOI:** 10.14202/vetworld.2020.2555-2564

**Published:** 2020-11-30

**Authors:** SukhDev Mishra, Rajendra Palkhade

**Affiliations:** ICMR-National Institute of Occupational Health, Ahmedabad, Gujarat, India

**Keywords:** accidents, needlestick, occupational hazards, physical hazards, veterinarians, workplace absenteeism, work-related injuries

## Abstract

**Background and Aim:**

Veterinary medicine is a high-risk occupation and imparts a risk of physical injuries due to the unpredictable nature of the animals and workplace conditions. This study aimed to identify the associated risk factors and prevalence of work-related injuries, and automobile accidents among veterinarians in India.

**Materials and Methods:**

A cross-sectional study was carried out among veterinarians (n=565). The responses were recorded using a self-administered questionnaire on work-related injuries, automobile accidents, and physical hazards.

**Results:**

Work-related injuries due to animals in the past 2 years were reported by more than half of veterinarians (prevalence=54.7%, 95% confidence interval [CI]=50.58-58.79), while two-thirds experienced workplace injuries due to animals during any time of their career. The risk for injury was 1.1 times higher (odds ratio=1.1, 95% CI=0.611, 1.981) for veterinarians with a long job duration (>10 years). Large animal practicing veterinarians faced a higher (2.03 times) risk of injury. Workplace absenteeism due to animal-related injury (up to 15 or more days) was reported by 25.9% (95% CI=22.44-29.68) of respondents, including hospitalizations for 7.8% of veterinarians. More than half of veterinarians suffered from automobile injuries (prevalence=60.9%, 95% CI=6.8-64.8) due to work-related travel in the past 2 years, resulting in workplace absenteeism for 56.2% (95% CI=51.46-60.97) of subjects. The prevalence of needlestick injury among veterinarians was very high and reported as 80.9% (95% CI=77.49-83.99). Recapping of needles significantly increased the risk of needlestick injury by 1.67 times.

**Conclusion:**

Veterinarians are at risk of work-related injuries, including automobile accidents. Kicking by animals and needlestick injuries were the most frequent physical hazards. Recapping of needles and responding to emergency calls at night were significant risk factors for needlestick injury and automobile accidents, respectively.

## Introduction

Veterinary professionals work in broad clinical settings, where varied working environments with varied kinds of species/patients are encountered [1]. The working environment and nature of the work itself are both important factors that can influence health [2]. In the process of diagnosis, treatment, vaccination, and providing veterinary care to livestock, veterinarians are exposed to occupational hazards in various forms. Animal health is one of the components of the “One Health” approach, whose realization depends prominently on the physical and psychological well-being of veterinarians.

The hazards in the field of veterinary medicine have been classified as physical, chemical, biological, and psychological [3]. Clinical and epidemiological studies have reported that veterinarians are at increased risk for different occupational-related illnesses [4,5]. Veterinary medicine is a high-risk group occupation in Australia [6]. The highest prevalence (64%) of trauma has been associated with large animal practice [4], which is more prone to physical injuries due to the large size and unpredictable nature of the patients [7].

In a Finnish study, the veterinary profession was described as physically demanding and linked to an elevated risk of accidents, ranging from moderate to considerably high, including the development of musculoskeletal disorders [8].

Needlestick injury cases have been reported in the literature ranging from low (22%) to high (58%) occurrences, sometimes resulting in days off work [9,10]. Similarly, in another study involving women veterinarians, the proportion of needlestick injury was as high as 63.9% [10].

India has one of the largest livestock sectors in the world, and animal husbandry is the second largest occupation in India. Livestock is more valued as a source of food, contributing to one-fourth of the agriculture gross and domestic product, and involving 9% of the agriculture workforce.

In India, many veterinarians work away from their clinic while performing their day-to-day work, especially in rural areas. Veterinarians need to travel long distances, which poses them venerable to automobile accidents, such as in case of emergency veterinary care to animals. Veterinarians in rural parts of the country are also involved in the implementation of government extension schemes for poverty alleviation, employment generation, and women’s empowerment by promoting animal husbandry activities. In society, veterinarians act as a bridge between human health and animal health in the framework of public health, for example, as counselors in dog bite cases. Veterinarians in Gujarat and Maharashtra state, India, and especially in the region where this study was carried out, predominantly involved in large animals practice. Animal husbandry is an important sector for the economy of the Gujarat and Maharashtra states (the human populations of Gujarat and Maharashtra states are 120.8 and 63.8 million, respectively). The previous studies conducted on occupational health hazards among Indian veterinarians have used small sample sizes and been confined to one area (few districts), such as wildlife or practice specific veterinarians [11], and physical injury and musculoskeletal disorders were noted among veterinarians in the three districts of Kerala state [9] as well as brucellosis [12]. In view of the above, a comprehensive report on the occupational health status of veterinarians is required. As of March 2019, the number of registered veterinarians in India was 68,680 [13], leading to a deficit in the estimated requirement of veterinarians in the country, which is disproportionate to the growing population of livestock [9,14]. The inadequacy of veterinary support in terms of basic infrastructure (hospitals and diagnostic labs) has put extra pressure on the working field veterinarians.

Occupational health hazards among veterinarians from developed countries have been studied and documented well, but a large gap of similar information still exists about veterinarians in India. Better identification of the occupational hazards and understanding of other related factors may allow strategy or policy-makers to make informed decisions for better work conditions of veterinary professionals in the country.

The objective of this study was to identify the associated risk factors and prevalence of work-related injuries and automobile accidents among veterinarians in India.

## Materials and Methods

### Ethical approval and informed consent

The study was approved by the Human Ethics Committee, ICMR-National Institute of Occupational Health, Ahmedabad vide Agenda No. 3.6-2015. Informed consent was obtained from all participants.

### Study tools and data collection

The data collection tool used for this study was a self-administered, structured questionnaire. A questionnaire was prepared by reviewing various important aspects of the reported literature in various parts of the world among veterinarians. The final questionnaire included questions based on field conditions and the work environment of veterinarians in India. The appropriateness and validity of the questionnaire were tested. The study was conducted in 2017-2018, and veterinarians (n=565) from different districts of Gujarat and Maharashtra state were selected as per their willingness to participate in the study. Before the inclusion of individual veterinarians in the study, the purpose of the study was described to each veterinarian, and their written consent was sought. Only those veterinarians who had veterinary work experience of at least 2 years were included in the analysis. Those who had <2 years of experience, were involved in teaching of non-clinical subjects at veterinary colleges, or were not involved in clinical practice were excluded from the analysis. The identity of subjects who participated in this study was not revealed at any point.

Data were collected on demographic information and physical hazards that is, injury due to automobile accident/s or due to animals and needlestick injury. Demographic data were collected on different variables, such as gender, age, the highest qualification acquired, and marital status. The types of species encountered during occupational activities were summarized in a different category for each veterinarian (large animals: Cattle, pigs, horses, sheep, and goats; small animals: Dogs, cats, domestic rodents, birds, and reptiles). Those who treated small ruminants (sheep and goat), large ruminants (cattle and buffalo), and horses were classified as “Large animal practitioners.” Those who practiced with dogs, cats, and other small pet animals, such as rats (laboratory animals), were categorized as “Small animal practitioners.” Those veterinarians who were involved with the treatment and management of wildlife or zoo animals were classified as “Wildlife/zoo animal practitioner.” Those veterinarians who treated both small and large animals were classified as “Mixed animal practitioner” in the questionnaire. Information was also collected about the coverage of accidental insurance.

Individual responses were categorized by the type of veterinary work as indicated by the participant: Private practice, academia/referral clinics, animal welfare organization, government, R&D, cooperative dairy societies, zoo/wildlife, and others. Most of the questions were closed, whereby participants were asked to select from a list of provided options.

For the assessment of physical hazards due to automobile accidents, detailed information, such as distance traveled for practice during work hours and mode of transportation, was sought. Frequencies of injuries caused by animals during the past 2 years and throughout the career were also recorded. Absence from work due to injuries caused by automobile accidents and due to animals was recorded. Physical injury due to animals was described by frequencies of injuries (e.g., 1-5 times or more instances) and covered under specific categories: Biting, scratches, dislocation, kicking, slip off, fracture, stamping on feet, crushing against wall, needlestick injury, and joint dislocation. Accident-prone individuals were defined as those who were admitted to hospitals for at least 24 h or the same duration of loss of working hours. At the same time, the occurrence of needlestick injuries, such as vaccination or general treatment during intravenous infusion, was recorded.

### Statistical analysis

Continuous variables, such as age or length of practice, are presented as the means±SD, while categorical variables are reported as the counts and percentage. The 95% confidence interval (CI) was computed for single proportions. Chi-square test and binary logistic regression analysis were carried out to assess the adjusted impact of independent variables on injury and automobile accidents in the form of an odds ratio (OR). The p<0.05 was regarded as statistically significant. All statistical analyses were carried out using the statistical software IBM SPSS Statistics for Windows, Version 26.0 (IBM Corp., Armonk, NY, USA).

## Results

### Demographic and professional characteristics

Among the total veterinarians interviewed (n=565), most subjects were male (90.4%), and female veterinarians comprised only 9.6%, with an overall mean age of 39.94±9.75 years; most of the subjects were married (93.5%). A few individuals (5.3%) had Ph.D. qualifications in veterinary sciences and chose to practice animal health, followed by 42.5% and 52.2% of graduates and postgraduates in different disciplines of veterinary science, respectively (Table-1). Almost three-fourths of the study population (74.3%) was government appointed veterinarians, while 12.6% of veterinarians were employed with different cooperative dairy societies. Large animal practitioners accounted for 60.3% of the total interviewed subjects. In contrast, small animal practitioners and mixed (both small and large animals) practitioners comprised 5.5% and 33.3%, respectively (Table-2).

**Table-1:** Demographic information of the study subjects.

Subject characteristics (n=565)	n (%)
Age (mean±SD) (years)	39.94±9.75
Gender	
Male	511 (90.4)
Female	54 (9.6)
Level of highest education	
Graduation	240 (42.5)
Postgraduation	295 (52.2)
Ph.D.	30 (5.3)
Marital status	
Married	528 (93.5)
Single	35 (6.2)
Separated	2 (0.4)

**Table-2:** Characteristics of workplace environment and category of animal practice.

Workplace characteristics	n (%)
Types of animal practices (n=565)	15 (2.7)
Private organization	15 (2.7)
Referral clinic	17 (3.0)
Government facility	420 (74.3)
Academic institute	19 (3.4)
Disease investigation laboratory	5 (0.9)
Research and development	2 (0.4)
NGO (welfare organization)	7 (1.2)
Dairy industry	71 (12.6)
Zoo practitioners	4 (0.7)
Others	5 (0.9)
Types of animal practices (n=559)	
Large animals	337 (60.3)
Small animals	31 (5.5)
Wildlife/zoo	5 (0.9)
Mixed (small and large) practice	186 (33.3)

### Physical hazards

#### Injuries due to animals

Although the majority of veterinarians worked with large animals, posing a high risk of work-related injury, few veterinarians (5.2%) were not aware of their insurance coverage. Out of all interviewed subjects, 54.7% suffered animal-related injuries (prevalence=54.7%, 95% CI=50.58-58.79) during the past 2 years. The frequency of injury differed among respondents; veterinarians who suffered injuries more than 10 times comprised 7.7% of the population, whereas injuries in the range of 5-10 times were reported by 22.6%. Nearly one-fourth (24.4%) of veterinarians suffered injuries 1-5 times. In addition, nearly 71% of the respondents mentioned animal-related injuries (up to 30 or more instances) during their careers.

Injuries due to animals also caused workplace absenteeism, and 25.9% (95% CI=22.44-26.68) of veterinarians had to remain absent from work for 1-15 days (or more) during the past 2 years (Table-3).

**Table-3:** Outcome distribution of animal injury related to veterinary practice.

Animal injury-related outcomes	n (%)	95% CI
Frequencies of injuries due to animals during the total career (n=562)		
5-15	259 (46.1)	(42.08, 50.30)
15-30	88 (15.7)	(12.91, 18.94)
30-more	51 (9.1)	(6.97, 11.78)
Never	164 (29.2)	(25.62, 33.13)
Frequencies of injuries due to animals during the past 2 years (n=561)		
1-5	137 (24.4)	(21.20, 28.34)
5-10	127 (22.6)	(19.51, 26.47)
10-more	43 (7.7)	(5.77, 10.26)
Never	254 (45.3)	(41.51, 49.75)
Animal biting (n=557)	
1-2	92 (16.5)	(13.66, 19.84)
2-5	73 (13.1)	(10.55, 16.18)
5-more	16 (2.9)	(1.75, 4.66)
Never	376 (67.5)	(63.50, 71.25)
Scratches (n=561)	
1-2	72 (12.8)	(10.31, 15.87)
2-5	116 (20.7)	(17.53, 24.23)
5-more	92 (16.4)	(13.56, 19.70)
Never	281 (50.1)	(45.96, 54.21)
Dislocation (n=560)	
1-2	23 (4.1)	(2.73, 6.13)
2-3	10 (1.8)	(0.93, 3.31)
3-more	4 (0.7)	(0.217, 1.91)
Never	523 (93.4)	(90.99, 95.17)
Kicking (n=560)	
1-2	101(18.0)	(15.07, 21.45)
2-5	113 (20.2)	(17.06, 23.71)
5-more	163 (29.1)	(25.50, 33.00)
Never	183 (32.7%)	(28.92, 36.67)
Slip off (n=560)	
1-2	61 (10.9%)	(8.57, 13.76)
2-5	100 (17.9%)	(14.90, 21.26)
5-more	90 (16.1%)	(13.26, 19.36)
Never	309 (55.2%)	(51.03, 59.24)
Fracture (n=557)	
1-2	41 (7.4%)	(5.46, 9.86)
2-3	16 (2.9)	(1.75, 4.66)
3-more	4 (0.7)	(0.21, 1.92)
Never	496 (89.0)	(86.15, 91.38)
Stamping on feet (n=559)	
1-2	67 (12.0)	(9.54, 14.96)
2-3	87 (15.6)	(12.79, 18.82)
3-more	105 (18.8)	(15.76, 22.24)
Never	300 (53.7)	(49.52, 57.76)
Crushing against the wall (n=557)	
1-2	81 (14.7)	(11.85, 17.73)
2-3	83 (15.1)	(12.18, 18.11)
3-more	52 (9.6)	(7.18, 12.06)
Never	341(61.1)	(57.10, 65.17)
Admitted to hospital due animal-related injury (during the past 2 years) (n=561)		
Yes	44 (7.8)	(5.88, 10.3)
No	517 (92.2)	(89.60, 94.11)
Absent from work due to animal-related injury (days) (during the past 2 years) (n=560)		
1-5	115 (20.5)	(17.39, 24.09)
5-15	14 (2.5)	(1.46, 4.20)
15-more	16 (2.9)	(1.74, 4.64)
Not admitted	415 (74.1)	(70.31, 77.55)
Occurrence of injury in case of place of treatment (n=559)		
Stable	33 (5.9)	(4.22, 8.21)
Field	398 (71.2)	(67.30, 74.79)
Dispensary	31 (5.5)	(3.92, 7.80)
Cattle camp	50 (8.9)	(6.84, 11.62)
Not applicable	47 (8.4)	(6.37, 11.03)
Occurrence of injury during the day (n=493)		
Morning hours	155 (31.5)	(27.5, 35.6)
Afternoon	148 (30.0)	(26.1, 34.2)
Evening hours	190 (38.5)	(34.3, 42.9)

CI=Confidence interval

#### Animal injuries in details

Animal biting

Nearly one-third (32.5%) of veterinarians reported multiple instances of animal biting injuries while treating animals (1-5 times or more). The risk of biting injury was higher among veterinarians with longer (>10 years) job duration (OR=1.009, 95% CI=0.539, 1.886).

Scratches

Of the total respondents surveyed, 12.8% got scratches (1-2 times) while treating animals. Moreover, 2-5 and >5 scratches due to animals were reported by 20.7% and 16.4% of respondents, respectively.

Kicking

Kicking by animals was mostly experienced by large animal veterinary practitioners. Nearly 29.1% of veterinarians had suffered animal kicking more than 5 times in the past 2 years of practice.

Slip off

Nearly 28.8% of veterinarians suffered from slip off during the treatment of animals up to 5 times in their routine work. Overall, 16.1% of veterinarians reported slipping off more than 5 times.

Fracture

Sixty-one individuals (11%) among total respondents reported a bone fracture (up to 3 or more instances) during animal treatment during the past 2 years. Longer job duration (>10 years) was associated with increased risk of fracture among veterinarians (OR=1.043, 95% CI=0.414, 2.723).

Stamped on feet

Veterinarians reported getting stamped on the feet by large animals while performing clinical examinations, vaccinations, or general treatment. Overall, 46.4% of the respondents experienced stamping by animal feet, and the frequency of stamping ranged from 1-2 times (12%), 2-3 times (15.6%), to >3 (18.8%) times.

Crushing against the wall

Of the total responding veterinarians, 39.4% experienced crushing against the wall by an animal on multiple instances. Crushing against the wall 1-2 times was reported by 14.7%, while 15.1% and 9.6% reported crushing against the wall 2-3 times or >3 times, respectively.

#### Dislocation

In total, 23 veterinarians suffered dislocation either 1-2 times (4.1%), 2-3 times (1.8%), or more than 3 times (0.7%).

Overall, severe animal-related injuries requiring hospitalization were reported by 7.8% of veterinarians during the past 2 years. Although not all respondents could recall the time of injury, most of the injuries (38.5%) occurred during evening work hours. A large number of veterinarians (71.2%) reported the field as a place of injury, while 8.9% reported getting injured at cattle camps.

#### Work-related automobile accidents

To attend animal patients, most veterinarians used a motorcycle (63.8%) followed by car (26.8%) and public transport (8.7%) as modes of transportation. Approximately 31% of the total respondents suffered automobile injuries more than 5 times, followed by 18.3% and 11.5% of subjects who suffered automobile injuries 1-3 and 3-5 times during the past 2 years, respectively. Only 53.6% of veterinarians had accidents insurance coverage in their self supported insurance policy. Approximately 53.1% of veterinarians remained absent from work due to automobile injuries, and the period of absence varied from 1-5 days (39.1%), 5-15 days (10.4%), to 15-30 days (3.6%). In contrast, more than 30 days of absenteeism was found among 3.1% of the total respondents. Overall, more than half of veterinarians suffered from automobile injuries (prevalence=60.9%, 95% CI=56.8-64.8) due to work-related travel in the past 2 years, which resulted in workplace absenteeism for 56.2% (95% CI=51.46-60.97) of subjects.

Work-related travel distance information from subjects revealed that 25%, 35.4%, and 3.6% of the total respondents traveled approximately 25-50 km, 50-100 km, and 100-150 km daily, respectively, to attend animal cases. Three-fourths (75.6%) of veterinarians among all respondents claimed that they attended emergency calls (1-10 calls/week) at night (Table-4). Further, risk analysis showed that automobile accident risk was 2.09 times higher (OR=2.09, 95% CI=1.321, 3.325, p<0.05) for veterinarians who attended night emergency calls for animal health care (Table-5).

**Table-4:** Distribution of occupational factors related to veterinary practice in reference to automobile injury.

Occupational characteristics	n (%)	95% CI
Attending emergency calls at night (per week) (n=563)		
1-2 calls	254 (45.1)	(41.05, 49.24)
3-5 calls	115 (20.4)	(17.30, 23.96)
6-10 calls	57 (10.1)	(7.89, 12.91)
No call	137 (24.3)	(20.97, 28.05)
Distance of travel while attending the case (n=559)		
1-10 km	88 (15.7)	(12.95, 19.01)
10-25 km	113 (20.2)	(17.09, 23.75)
25-50 km	140 (25.0)	(21.63, 28.80)
50-100 km	198 (35.4)	(31.57, 39.47)
100-150 km	20 (3.6)	(2.3, 5.58)
Mode of transportation (n=563)		
Motorcycle	359 (63.8)	(59.70, 67.62)
Car	151 (26.8)	(23.33, 30.63)
Public transportation	49 (8.7)	(6.63, 11.34)
Walking	4 (0.7)	(0.215, 1.90)
Frequencies of automobile accidents during the past 2 years (n=563)		
1-3	103 (18.3)	(15.32, 21.71)
3-5	65 (11.5)	(9.15, 14.47)
5-more	175 (31.1)	(27.4, 35.03)
No accident	220 (39.1)	(35.13, 43.17)
Workplace absenteeism in days (during the past 2 years) (n=414)		
1-5	162 (39.1)	(34.55, 43.91)
5-15	43 (10.4)	(7.79, 13.73)
15-30	15 (3.6)	(2.17, 5.96)
30-more	13 (3.1)	(1.8, 5.37)
No absenteeism	181 (43.7)	(39.02, 48.53)
Whether covered by any accident insurance (n=562)		
Yes	301 (53.6)	(49.42, 57.64)
Group insurance scheme	59 (10.5)	(8.22, 13.33)
No	173 (30.8)	(27.11, 34.72)
Not aware	29 (5.2)	(3.60, 7.35)

**Table-5:** Risk factor analysis for animal caused injuries and automobile accidents.

	Yes (%)	No (%)	Odds ratio (adj.) (95% CI)
Factor: Length of career as practicing veterinarian			
Injury in whole career			
0-10 years	143 (55.2)	116 (44.8)	1.1 (0.611, 1.981)
>10 years	164 (53.6)	142 (46.4)	
Biting by animal*			
0-10 years	71 (27.95)	183 (72.05)	1.009 (0.539, 1.886)
>10 years	110 (36.79)	189 (63.21)	
Scratches by animal			
0-10 years	128 (49.81)	129 (50.19)	0.9 (0.501, 1.619)
>10 years	152 (50.33)	150 (49.67)	
Kicking			
0-10 years	179 (69.65)	78 (30.35)	0.991 (0.529, 1.857)
>10 years	198 (66)	102 (34)	
Fracture by animal*			
0-10 years	245 (94.59)	14 (5.41)	1.043 (0.414, 2.723)
>10 years	279 (91.18)	27 (8.82)	
Slip off			
0-10 years	118 (45.91)	139 (54.09)	0.928 (0.514, 1.677)
>10 years	133 (44.33)	167 (55.67)	
Stamping on feet			
0-10 years	120 (46.69)	137 (53.31)	0.981 (0.544, 1.771)
>10 years	139 (46.49)	160 (53.51)	
Crushing against the wall			
0-10 years	106 (41.57)	149 (58.43)	1.146 (0.625, 2.101)
>10 years	110 (36.79)	189 (63.21)	
Hospitalization			
0-10 years	244 (94.21)	15 (5.79)	0.692 (0.228, 2.098)
>10 years	280 (91.5)	26 (8.5)	
Factor: Type of animal practice			
Animal size			
Large	151 (44.3)	190 (55.7)	2.033 (0.954, 4.332)
Small	19 (61.3)	12 (38.7)	
Factor: Needle destruction method			
Method of destruction			
Recapping	91 (87.5)	13 (12.5)	1.672* (1.074, 2.604)
Biowaste/disposal	361(79.9)	91 (20.1)	
Factor: Attending night calls for veterinary emergency and automobile accidents			
Night calls*			
Yes	152 (35.85)	272 (64.15)	2.096* (1.321, 3.325)
No	28 (21.05)	105 (78.95)	

* p<0.05 (χ^2^ test), OR is based on univariate binary logistic regression adjusted for job length. OR=Odds ratio

#### Needlestick injury

During veterinary practice, many veterinarians (80.9%) reported suffering a needlestick injury (95% CI=77.49-83.99). The injuries happened multiple times in the previous 2 years, and the present survey findings revealed that 26.3% of veterinarians suffered from needlestick injuries more than 10 times. Overall, 34.0% of respondents received between 5 and 10 needlestick injuries. Although vaccination (25.2%) and intravenous infusion (2.9%) are common procedural reasons for needlestick injuries among veterinarians, our study findings show that the largest proportion of veterinarians got injured during the general treatment of animals (51.9%).

Respondents were asked about their needle disposal method to better understand their needlestick injury. Overall, 18.9% of veterinarians practiced the recapping of needles after use, while 23.5% disposed of the used needles through a biowaste agency. Nearly half of the respondents (49.9%) mentioned the use of a disposable box for disposing of needles (Table-6). The risk of needlestick was significantly higher (1.67 times) among veterinarians who recapped needles for disposal (OR=1.67, 95% CI=1.074, 2.604) (Table-5).

**Table-6:** Needlestick injury among veterinarians during animal practice.

Injury characteristic	n (%)	95% CI
Needlestick injury during the past 2 years (n=562)		
1-5	116 (20.6)	(17.50, 24.19)
5-10	191 (34.0)	(30.19, 38.00)
10-more	148 (26.3)	(22.86, 30.13)
Never	107 (19.0)	(16.00, 22.50)
Occurrence of needlestick injury (n=547)		
Vaccination	138 (25.2)	(21.77, 29.04)
General treatment	284 (51.9)	(47.73, 56.07)
Intravenous infusion	16 (2.9)	(1.78, 4.75)
Cannot say	109 (19.9)	(16.79, 23.49)
Destroying of used needles (n=557)		
Recapping of needles	105 (18.9)	(15.82, 22.32)
Needle disposable box	278 (49.9)	(45.77, 54.04)
Biowaste agency	131 (23.5)	(20.18, 27.22)
Do not know	43 (7.7)	(5.77, 1.02)
Self-medication (n=556)		
Yes	252 (45.3)	(41.23, 49.48)
No	46 (8.3)	(6.25, 10.89)
Do not want to disclose	258 (46.4)	(42.29, 50.55)

#### Self-medication

To explore the possibility of self-medication by veterinarians, the study asked respondents about self-medication, and the findings revealed that 45.3% of veterinarians admitted self-medication in case of injury or illness (Table-6).

## Discussion

There has been a wide information gap regarding occupational health hazards and accidental injuries among Indian veterinary professionals, as previous reports of occupational health risk among veterinarians have been limited by inadequate numbers of subjects [9,11].

The distribution of respondents showed that the veterinary profession is largely male. Male dominance in the veterinary profession can be explained by educational and demographical differences by regions in India. In the present study, the proportion of female practitioners was higher (9.6%) than an earlier reported study [11] from the North region of India. Pillai [9] reported a larger percentage of women veterinary professionals (38.9%) than the present finding; this wide difference within the country may be attributed to the highest literacy rate among women and the highest female-to-male ratio (1084/1000) in Kerala than other Indian states. A study conducted on the USA swine practitioners [15] revealed male dominancy in the profession, constituting 93.8% over female (6.2%) practitioners. In the USA, more than 50% of practicing veterinarians are women, whereas practicing female veterinarians comprise 43% in Canada [16]. Recently, there has been an increase in the number of females becoming admitted to the veterinary profession. It is estimated that there are more than 3000 female veterinarians in the country from different states registered with the Veterinary Council of India [17]. A major proportion of the present study subjects consisted of large animal practitioners (60.3%), which was also found to be a significant risk factor for injuries caused by animals. A similar finding was also reported by Thigpen and Dorn [18], where most animal-related injuries were related to large animal practitioners. Greater rates of trauma (64%) have also been associated with large animal practice [4].

An important objective of the study was to understand the nature and pattern of physical injuries in the study population. Previously, a large number of veterinarians suffered work-related musculoskeletal pain and discomfort that could be caused by physical and psychosocial risk factors [19,20]. Kicking and slip-off are exclusively associated with the handling of large animals, for example, cattle. In the present study, kicking (67.3%), scratches (49.9%), crushing against the wall (39.4%), and slip off (44.9%) were the most common injuries among the study population. Similar to the present study findings, kicks (35.5%), bites (34%), crushing (11.7%), and scratching (3.8%) were reported to be major injuries in veterinarians caused by animals [21]. Veterinarians and their associated workers were more prone to work-related injuries due to the unpredictable and uncooperative nature of animals. The equipment used for animal handling and different veterinary procedures is also a reason for injury among veterinarians. Trauma in the field of veterinary medicine may be attributed to bites, scratches, crushing against the wall, lifting, repetitive motion, motor vehicle accidents, assault, and scalpel cuts [6,22]. It has been reported that animal-related injuries are the major hazards (44%) among the wildlife veterinary occupation in India [11]. Needlestick (22%) was documented as the most frequent injury by Pillai [9].

Nienhaus *et al*. [23] reported that approximately 66% of work-related accidents were due to scratches, bites, or kicks from animals among veterinarians. Biting and scratches from pets, cats, and dogs are also potential sources of contracting zoonotic infections, for example, rabies and cat scratch disease (*Bartonella henselae*) [24,25]. A high rate of injuries (40-46.6%) was reported among veterinarians working with large animals [1,21], which underscores the importance of restraining animals and training associated staff. In the present study, 71.2% of respondents mentioned field/farm as the place of injury. Lucas *et al*. [1] reported animal farm as the place of injury of veterinarians in 55% of cases, which might be due to inadequate restraining equipment and support staff. We found that more than half of the studied population (54.7%) suffered a significant injury due to animals in the past 2 years. Pillai [9] also reported similar findings (47.6%) among Indian veterinarians, while a study conducted by Lucas *et al*. [1] among Australian veterinarians reported animal-related injuries among 26% of respondents. In our study, 70.9% of veterinarians suffered significant injuries during their career, which closely corroborates the findings (64.6%) of a study conducted by Lessenger [21] in Minnesota and Wisconsin in the United States. Another study from the United States reported animal-related major injuries among 61.1% of swine practitioner veterinarians [26]. Significant work-related injuries during the career were comparatively fewer among Australian veterinarians (51%), as reported by Fritschi *et al*. [7]. Our definition of a significant injury required hospitalization or at least 1 day of absence from work [27]. In our study, 7.8% of respondents had been admitted to the hospital for a work-related injury, which is close to the findings of Langley *et al*. [28] who reported hospitalizations among 8.2% of veterinarians in North Carolina. Absenteeism (at least one event of absence) from work was reported by 25.9% of study subjects, which was less than earlier reported absenteeism among Indian veterinarians (45.6%) [9]. Further, the reported workplace absenteeism in Canadian veterinarians was also less (17%) in comparison to Indian studies [10]. Animal biting injuries among veterinarians have been reported to be fatal [23], and our study revealed that 32.5% of Indian veterinarians had suffered a biting injury while treating animals (5 or more instances). In contrast, Nigam and Srivastav [11] found comparatively fewer (20.3%) biting injuries. Studies from the United States and Australia by Fritschi *et al*. [7] and Wiggins *et al*. [4] found the percentage of respondents suffering biting injuries to be 26% and 17%, respectively. This observed difference among studies could be attributed to the prevalent type of animal practice, either domestics/wild or small/large. Personal protective equipment adherence may prove critical in minimizing the risk of injury during large animal veterinary practice [29].

Veterinarians have to travel long distances to deliver animal health-care services at remote or rural sites to attend emergency calls. In this study, 35.9% of the field veterinarians were traveling 0-25 km daily, which is comparatively less than a study reported earlier in India [9]. Studies from other countries have reported veterinarians traveling more than 68 km/day [21], 79 km/day [6], and approximately 105 km/day among the US swine practitioners [15]. Direct comparisons about daily traveling by veterinarians while attending cases in different countries may not be conclusive because of geographical variations and the diversity of practices in veterinary medicine. A high population density could be considered an explanatory factor for the relatively decreased work-related travel in comparison to the United States and Australia, as the population density in India is more than 4 times higher than the other two countries [30]. Our study found a significant association along with higher risk (2.09 times) for attending emergency calls at night and suffering automobile accidents among veterinarians. A recent study by Irwin *et al*. [31] suggested an increased likelihood of risk and hazards for veterinarians responding to emergency calls during night-time driving and due to adverse weather conditions.

The data in the present study revealed that approximately 31.1% (>5 times), 11.5% (3-5 times), and 18.3% (1-3 times) of respondents suffered an automobile accident or vehicular injury while commuting for job/attending cases during the past 2 years. Among the United States veterinarians, this prevalence was reported as 28% [15], while automobile accidents in a Turkish study were encountered by 54.3% of veterinarians [32]. Nearly 38% of Finnish veterinarians considered accidents as a significant risk in the veterinary profession [8]. The present study revealed that the majority (63.8%) of veterinarians used a motorcycle for work-related commute, and only 26.8% of veterinarians had a car for work-related commutes. Extensive travel by veterinarians due to work poses an inherent accidental risk and has been covered by insurance in many countries [3,23,33] as an essential benefit. However, accidental insurance coverage among Indian veterinarians is less common (53.6%), and employer support for accidental coverage was mostly missing.

Needlestick injuries are common in veterinary practice, and preventive approaches as for human medicine are lacking [34]. Multiple common reasons for needlestick injuries have been reported, such as vaccinations, recapping of needles, performing surgery, and injecting drugs into animals [35,36]. Needlestick injuries may involve the risk of autoinoculation (e.g., antineoplastic drug and live vaccine). Other harmful substances, such as microorganisms, can result in allergic reactions or more harmful consequences, especially contracting either subclinical or fatal zoonotic infections. The present study revealed that the majority (80.9%) of respondents suffered from 1 to more than 10 needlestick injuries during the past 2 years. Epp and Waldner [10] reported that 58% of veterinarians suffered needlestick injuries in West Canada. Similarly, 73% of US swine practitioners experienced a needlestick injury at least once in their career [15]. Mshelbwala *et al*. [34] found that 79.5% of Nigerian veterinarians had suffered a needlestick injury. Needlestick injury can range from local inflammation to fatal illness depending on the substance contained in the syringes at the time of the puncture event. Accidental self-injection of prostaglandin through a needlestick injury may also lead to spontaneous abortion (in the case of a pregnant female veterinarian), resulting in a serious human reproductive health hazard [3]. Both small and mixed animal practicing veterinarians demonstrated high rates of needlestick injury [37]. In the present study, recapping of needles, which has been identified as a significant risk factor for needlestick injury, was reported by 18.9% of the respondents. Furthermore, nearly 45.3% of the respondents agreed that they self-medicated in case of injury or illness. Landercasper *et al*. [27] also reported a similar finding that two-thirds of veterinarians were self-treated for injuries and illness. Lucas *et al*. [1] reported that veterinarians preferred self-medication (23%) in their study. In the present study, the time of occurrence of injuries was reported by 38.5% of the veterinarians as evening, in contrast to working in afternoon was reported by Canadian veterinarian as a risk factor [10].

More recently, to deal with the above occupational health hazards in the workplace among veterinarians, more comprehensive well-being strategies have been echoed by researchers for adoption by organizations and industries [2,38].

## Conclusion

The study findings indicate that practicing veterinary professionals in India face various kinds of occupational health hazards. Prominent injuries reported by veterinarians include needlesticks, automobile accidents, and animal kicking. Recapping of needles and responding to emergency calls at night were significant risk factors for needlestick injury and automobile accidents, respectively.

Although work-related automobile injuries were experienced by a large number of respondents, accidental insurance coverage among Indian veterinarians is low. We opine that insurance should be mandatory coverage for veterinary professionals by employers. Policy level changes, such as proper training and induction to safe practicing guidelines at the job entry-level, can be helpful in reducing needlestick injuries among veterinarians. Adopting safe methods of needle disposal should be encouraged instead of the recapping method, which was prevalent among the studied subjects. Proper restraining of animals and other appropriate facilities can greatly reduce the risk of physical injury among veterinarians, which requires proper Occupational Health and Safety (OHS) training for veterinarians as well as to their associate staff. We suggest including an OHS curriculum in Indian Veterinary Medicine schools.

### Limitation of the study

This study was conducted under a cross-sectional design and recorded the responses of practicing veterinarians through a questionnaire in a self-assessment manner. The information provided by the study subjects on past events could be a potential source of recall bias.

## Authors’ Contributions

RP designed and conducted the experiments. RP and SM analyzed the results. RP and SM drafted and reviewed the manuscript. Both authors read and approved the final manuscript.
